# Internal tides can provide thermal refugia that will buffer some coral reefs from future global warming

**DOI:** 10.1038/s41598-020-70372-9

**Published:** 2020-08-10

**Authors:** Curt D. Storlazzi, Olivia M. Cheriton, Ruben van Hooidonk, Zhongxiang Zhao, Russell Brainard

**Affiliations:** 1 U.S. Geological Survey, Pacific Coastal and Marine Science Center, 2885 Mission Street, Santa Cruz, CA 95060 USA; 2 Cooperative Institute for Marine and Atmospheric Studies, University of Miami, Miami, FL 33149 USA; 3 Applied Physics Laboratory, University of Washington, Seattle, WA 98105 USA; 4 King Abdullah University of Science and Technology, Red Sea Research Center, Thuwal, Saudi Arabia

**Keywords:** Climate-change ecology, Climate-change impacts, Projection and prediction, Marine biology, Physical oceanography

## Abstract

Observations show ocean temperatures are rising due to climate change, resulting in a fivefold increase in the incidence of regional-scale coral bleaching events since the 1980s; analyses based on global climate models forecast bleaching will become an annual event for most of the world’s coral reefs within 30–50 yr. Internal waves at tidal frequencies can regularly flush reefs with cooler waters, buffering the thermal stress from rising sea-surface temperatures. Here we present the first global maps of the effects these processes have on bleaching projections for three IPCC-AR5 emissions scenarios. Incorporating semidiurnal temperature fluctuations into the projected water temperatures at depth creates a delay in the timing of annual severe bleaching ≥ 10 yr (≥ 20 yr) for 38% (9%), 15% (1%), and 1% (0%) of coral reef sites for the low, moderate, and high emission scenarios, respectively; regional averages can reach twice as high. These cooling effects are greatest later in twenty-first century for the moderate emission scenarios, and around the middle twenty-first century for the highest emission scenario. Our results demonstrate how these effects could delay bleaching for corals, providing thermal refugia. Identification of such areas could be a factor for the selection of coral reef marine protected areas.

## Introduction

One of the major causes of mass coral reef bleaching events worldwide is rising sea temperatures caused primarily by anthropogenic global warming^1–8^. Bleaching can occur when corals that are exposed to sustained temperatures above their average annual ranges expel their symbiotic algae. Although it is possible for bleached corals to recover, this process can take many years or decades and the recovered reefs will likely have initially reduced species diversity with fewer reef-building coral species^9–11^. When these bleaching events occur annually, it is unlikely that heat-sensitive coral species can survive, as this frequency of bleaching does not give the corals sufficient time to recover^12,13^.

Remotely sensed sea surface temperatures (SSTs) have been used as a tool for monitoring the global ocean for anomalously high temperatures and evaluating current and potential future bleaching threats. The National Oceanographic and Atmospheric Administration’s (NOAA) Coral Reef Watch (CRW) program (https://coralreefwatch.noaa.gov) developed a coral bleaching monitoring and prediction system based on satellite SST measurements, modeling, and in situ measurements^14^. Accumulating heat stress is quantified using degree-heating weeks (DHW) and severe bleaching conditions have been correlated with DHW > 8 °C-weeks^15^.

More recently, Global Climate Model (GCM) projections of SST have been used to predict when different regions will start to experience severe coral bleaching often enough that they will not recover, finding that with “business as usual” emissions^16,17^ (RCP8.5), bleaching as modeled using accumulated heat stress via DHW will become an annual event for most of the world’s coral reefs within 30–50 yr^3,4,18,19^. One critical source of uncertainty in such projections is the large difference between the horizontal scale of individual coral reefs (10^1^–10^4^ m) and the scale of coupled atmosphere–ocean GCMs used to project future climate^16–18^ (10^4^–10^5^ m).

In addition to the disparity in spatial resolution of the GCMs relative to the scales of reef communities, this coarse spatial and temporal resolution cannot capture meso- and sub-mesoscale processes that can also affect the exposure of corals to anomalous temperatures, such as upwelling, diurnal heating, eddies, and internal waves. Many of these processes lead to strong vertical thermal gradients, whereby the ocean temperature near the benthic substrate, where the corals reside, is cooler than at the surface. In particular, internal waves at the tidal frequency and greater can advect deeper, cooler (~ °C) waters up (~ 10 s–100 s m vertically and ~ 100 s–1000 s m horizontally) into shallower-water environments. These features have been shown to occur over shallow-water, hermatypic (0–40 m depths) coral reefs worldwide^20–25^. Because internal waves regularly flush reefs with cooler waters, they can modify species assemblages^25^, reduce thermal stress^26–29^ and corals’ susceptibility to bleaching^30^.

The bleak outlook on coral reef futures underscores the importance of identifying places where processes that buffer the thermal stress from rising SSTs may serve as thermal refugia. Here, we present the first attempt to quantify the effects internal wave processes might have on moderating future thermally induced bleaching for coral reefs globally. We quantify the spatial and vertical patterns of semi-diurnal internal waves’ influence on water temperatures over coral reefs, determine how those patterns will vary in the future for different climate-change scenarios, and discuss how such information may be incorporated into the decision-making process for designating Marine Protected Areas (MPAs) for coral reef protection and preservation.

## Materials and methods

Semidiurnal temperature fluctuations were constructed using past climatology of ocean vertical thermal structure coupled with a global dataset of semidiurnal internal tide amplitudes; they were then applied to projected SSTs for different climate change scenarios. Here, “semidiurnal” is used to mean the principal lunar (M2; period ~ 12.42 h) and the principal solar (S2; period ~ 12.00 h) semidiurnal tidal constituents. We note that, because we lack full water-column temperature, we cannot delineate barotropic from baroclinic semidiurnal internal tide driven temperature variability; as such, we refer to these patterns as “semidiurnal temperature fluctuations.” The objective of our analyses was to allow for direct comparison with the findings of van Hooidonk et al.^18^. Details are provided below on the model and observational datasets used in the analyses.

### Model of semidiurnal temperature fluctuations

The global coral reef locations (1° × 1° pixels) were obtained from the merged reefbase/UNEP-WCMC and Millennium Coral Reef Mapping Project reefs database (https://imars.usf.edu/MC/index.html). For each coral reef location (1° × 1° pixel), we found the monthly climatology of vertical ocean temperature using the Argo float dataset^31^. These data are collected and made freely available by the International Argo Program and the national programs that contribute to it (https://www.argo.ucsd.edu, https://argo.jcommops.org). The Argo Program is part of the Global Ocean Observing System. The Roemmich-Gilson Argo climatology (https://sio-argo.ucsd.edu/RG_Climatology.html) provides a gridded, optimally interpolated, upper-ocean temperature climatology and monthly anomaly field^32^ that continues to be updated; the data utilized here were for 2004–2016, with 58 vertical steps down to 2000 dbar.

**Figure 1:**
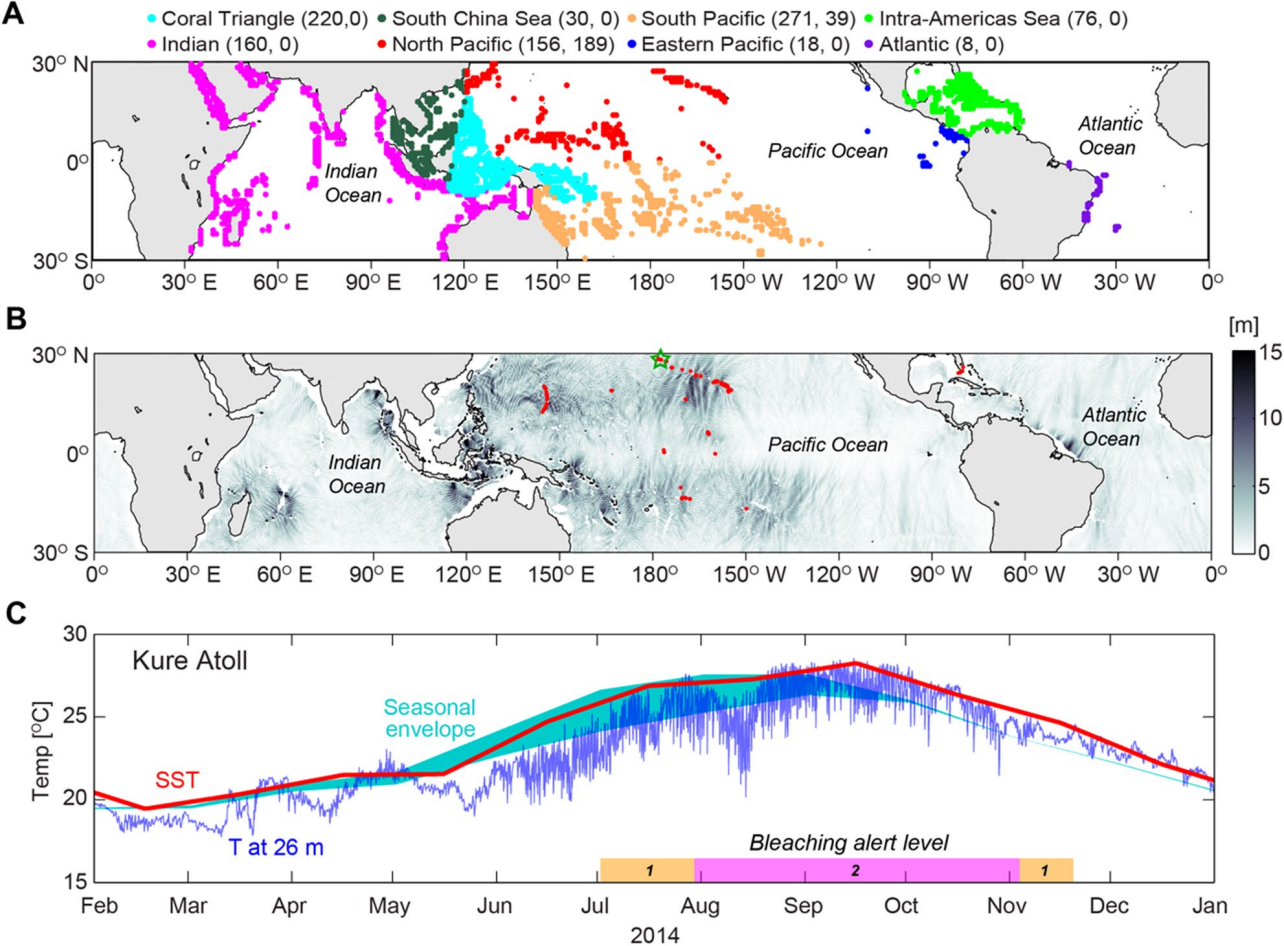
Locations of coral reefs, internal tide amplitude, and example of in situ and modeled data from a reef site. (**A**) Map showing the locations of coral reefs and the geographic regions defined here from the merged reefbase/UNEP-WCMC and Millennium Coral Reef Mapping Project reefs database (https://imars.usf.edu/MC/index.html), with the total number of reef sites and the number of in situ observations for each region in parentheses. (**B**) Map showing the Zhao et al. (2016) open ocean, mode-1, semidiurnal (M2 + S2) internal tide amplitude in gray shading, with the 233 in situ temperature sites overlaid (red dots). The green star shows the location of a representative site on Kure Atoll in the Northwestern Hawaiian Islands. (**C**) The in situ temperature (‘T at 26 m’) from the Kure Atoll site and the co-located Pathfinder sea surface temperature (SST) measured in 2014, when extensive coral bleaching occurred in many locations in the NWHI (Couch et al. 2017); the seasonal envelope from the mean monthly climatology is displayed with cyan shading, with the upper bound being SST and the lower bound the temperature at the depth given by the site depth plus the model amplitude. NOAA’s Coral Reef Watch NWHI virtual monitoring station bleaching alerts are shown along the bottom of **C**, where level 1 (orange) indicates significant bleaching is expected and level 2 (pink) indicates wide-spread bleaching and coral mortality are expected. Throughout the extensive bleaching event, semidiurnal-period fluctuations drove temperature decreases of up to 4 °C. The synthetic semidiurnal temperature has a smaller overall temperature decrease but systematically more chronic exposure.

**Figure 2:**
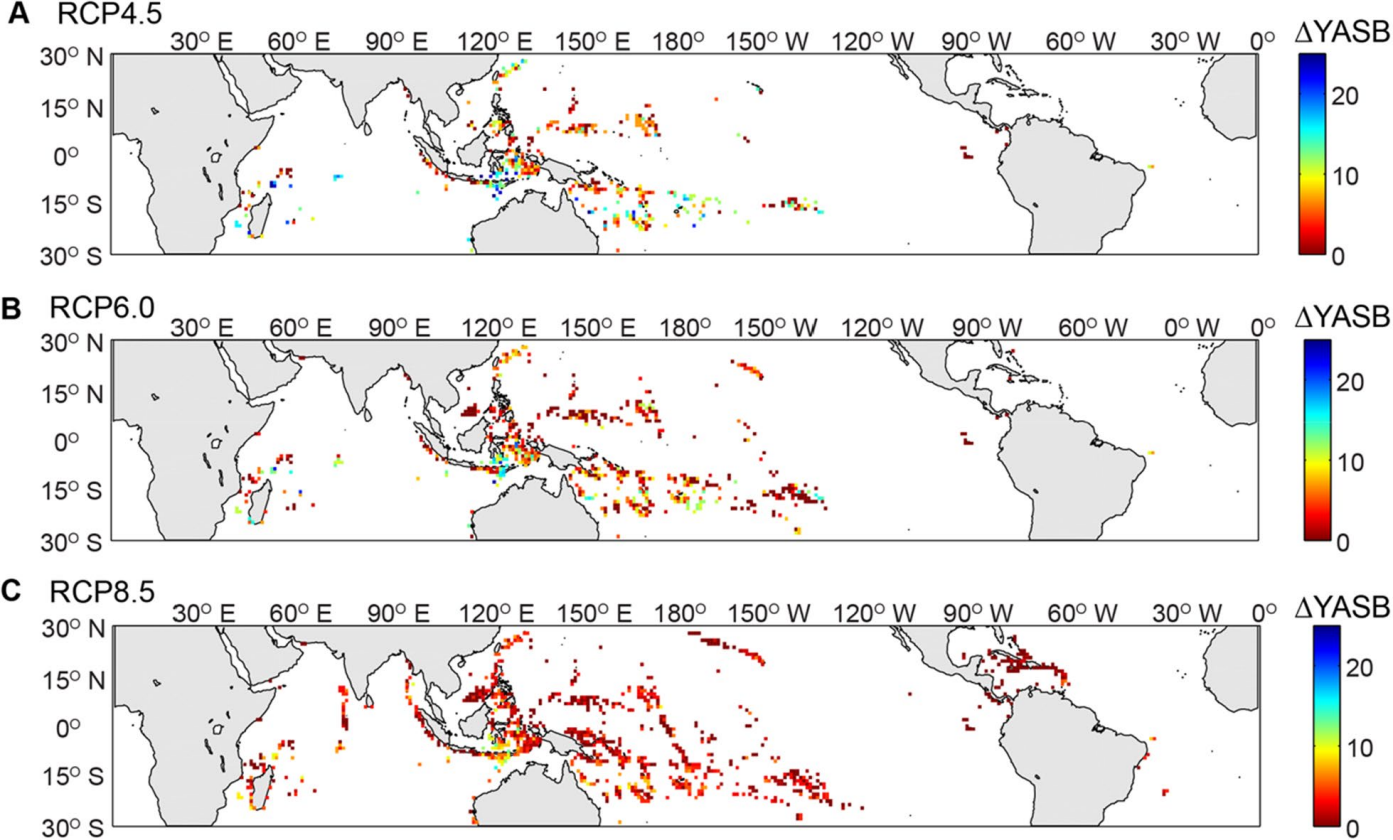
Map showing coral reef locations and the projected delay (∆) in the Year of Annual Severe Bleaching (YASB) for different Representative Concentration Pathway (RCP) scenarios. (**A**) RCP4.5. (**B**) RCP6.0. (**C**) RCP8.5. ∆YASB is the difference between YASB for sea-surface temperature (SST) projections and YASB with site-specific semidiurnal (M2 + S2) temperature fluctuations for coral reef sites at 20-m depths. Sites that are outside the range of the global, mode-1 internal tide observations (Zhao et al. 2016) and sites that never reach YASB_SST_ by 2090 are omitted. For many sites, ∆YASB attributed to semidiurnal temperature fluctuations is small (< 10 yr); however, for some reefs, the delay can be on the order of decades. The thermal benefit provided by the subsurface temperature fluctuations decreases for higher emission scenarios such as RCP8.5.

Next, a corresponding internal tide amplitude was found for each coral reef site using a global open-ocean mode-1 M2 and S2 internal tide dataset provided by Zhao et al.^33^. These amplitudes were computed based on sea surface height (SSH) measurements from multiple satellites. These observations only capture the coherent internal tide, missing the incoherent component, which may constitute 30–50% additional internal tide energy, especially close to the equator^34^. To partially account for this missing energy, the M2 and S2 amplitudes were increased by 30%. The associated semidiurnal internal tide amplitude used for each site was the mean within a 1° × 1° box centered on each coral reef pixel. Only pixels that contained at least 25% non-empty internal tide amplitude observations were used; this criterion allowed for 52% of the global coral sites (compare sites shown in Figs. 1a, 2b). Coral sites that lack internal tide amplitude information are primarily locations on broad continental shelves, such as those around Florida, USA, and the Bahamas, the Sunda shelf in Southeast Asia, the Arafura Shelf between Australia and Papua New Guinea, and the Red Sea. We also note that open-ocean internal tides can amplify as they propagate from deep to shallow waters, sometimes inducing internal solitary waves that pump cooler-deeper waters towards the surface^35^. Thus, these internal tide amplitudes likely represent an underestimation of the vertical internal motions in the nearshore.

The temperature decrease caused by the semidiurnal fluctuations was identified in the Argo monthly climatology as the difference between the SST and the temperature at a depth equal to the coral’s depth plus the internal tide amplitude for that location. Three coral reef depths were considered: 10, 20, and 30 m. The monthly temperature decrease was interpolated to 12-h intervals, and the yearly semidiurnal temperature fluctuation model pattern was this temperature decrease alternating with zero temperature decrease from SST. In the natural environment, nearshore internal waves do not necessarily result in thermal swings that approach sea surface values; often, their maximum temperatures are less than SSTs. In this respect, our model could be considered a conservative representation of potential cold-water exposure due to semidiurnal internal tides.

### Projected sea-surface temperature

The modeled semidiurnal temperature fluctuations for the global coral reef sites were applied to projected global SST timeseries from Van Hooidonk et al.^18^. These SST projections were taken from the World Climate Research Programme’s CMIP5 datase^5^ (see van Hooidonk et al.^18^ for complete table), and here we focus on the projected SST from three Representative Concentration Pathways (RCP) emission scenarios^16,17^: (1) RCP4.5, which is stabilization without overshoot, (2) RCP6.0, which is stabilization at a higher radiative forcing without overshoot), and (3) RCP8.5, which is fossil-fuel aggressive (i.e., baseline scenario). For each pixel, the projected SST time series was adjusted such that the 2006–2011 model mean was equal to the mean of the corresponding 1985–2005 Pathfinder SST (see section below). In addition, the annual cycles were replaced with those from the observed Pathfinder climatology. Missing pixels near the coasts were filled using a Poisson relaxation interpolation.

### Model evaluation

The modeled semidiurnal temperature fluctuations were compared to 225 subsurface, in situ, long-term temperature records collected at coral reef sites across the Pacific Ocean and Florida (Fig. 1a; see Supplementary Table S1 online). These subsurface data came from two sources: (1) the National Oceanic and Atmospheric Administration’s (NOAA) Pacific Islands Fisheries Science Center Coral Reef Ecosystem Program, made available through the National Coral Reef Monitoring Program^36^ (https://data.nodc.noaa.gov), and (2) the Coral Reef Research Foundation (https://coralreefpalau.org) long-term monitoring program around Palau. The locations of these measurements were primarily from fore reefs in depths between 10 to 30 m, and were from 47 distinct islands, atolls, submerged reefs, and coastal areas. Sites within lagoons and/or located > 5 km from the 50-m isobaths (i.e., a broad shelf separating them from the open ocean) were excluded. Temperatures were measured at intervals ranging from every 5 min to hourly and each record had a length of at least 2 yr (maximum length was 6.2 yr, mean = 2.2 yr). The corresponding SST time series for each site location and record time period were found using the Pathfinder Advanced Very High Resolution Radiometer (AVHRR) v4.1 + Global Area Coverage (GAC) monthly temperature (see Supplementary Table S1 online). This global SST has a 0.1° × 0.1° resolution and is available from the NOAA OceanWatch Central Pacific Node website (https://oceanwatch.pifsc.noaa.gov/).

At each of these in situ sites, the subsurface temperature was band-pass filtered (9.6–14.4 h) and the largest temperature decrease from SST was identified and compared to that from the modeled fluctuations (see Supplementary Fig. S1 online). There was a robust linear correlation (*r* = 0.81, *p* ≪ 0.05, *df* = 186) between the observed and the modeled maximum subsurface temperature, with a slope close to 1 (*m* = 1.3) and an offset of 0.5. Thus, the modeled and observed maximum subsurface temperature decrease roughly scale together and this linear regression was used to adjust the modeled time series for each global coral reef location.

### Year of annual severe bleaching (YASB)

Within the projected temperature time series, bleaching was defined using the degree-heating-weeks (DHW) metric^37^. As in van Hooidonk et al.^18^, DHW start to accumulate when projected temperatures exceed the maximum monthly mean (MMM) from the pathfinder climatology. Here, the MMM for each coral location is adjusted for the three considered depths (10, 20, and 30 m) using the mean temperature change with depth from the Argo climatology; this was done under the assumption that corals at deeper depths have reduced thermal tolerance. Severe bleaching with high mortality have been observed to occur when DHW > 8 °C-weeks^14,15^. For a coral reef site, the Year of Annual Severe Bleaching (YASB) is defined as the projected year when severe bleaching starts to occur at least once a year, for 10 consecutive yr^18^.

## Results

Per van Hooidonk et al.^18^, the YASB can be considered the “tipping point” for a coral reef, a conservative estimate of when recovery from consecutive bleaching events will no longer be possible. Here we use the RCP6.0 scenario as a baseline to compare and contrast the two end-member scenarios (RCP4.5 with drastic emission cuts and the RCP8.5 with unabated emission growth). With RCP6.0, using SST alone, 69% of coral reef sites with a corresponding internal tide amplitude are projected to reach YASB before 2091 (see Supplementary Fig. S2 online). Of these coral reef sites, imposing the effects of semidiurnal, internal-tide driven temperature fluctuations on the projected SST creates a delay in the timing of YASB (∆YASB) of ≥ 10 yr for 12–17% of sites, depending on depth, and ∆YASB ≥ 20 yr for up to 1% of sites (Fig. 2). Under a low emissions scenario (RCP4.5), ∆YASB afforded by the semidiurnal temperature fluctuations is greater, with 30–44% of sites experiencing a delay ≥ 10 yr and 8–10% of the sites experiencing a ∆YASB ≥ 20 yr. Under a high emissions scenario (RCP8.5), a ∆YASB ≥ 10 yr only transpires for up to 1% of sites at 20 m depth and no sites experiencing a ∆YASB ≥ 20 yr.

**Figure 3:**
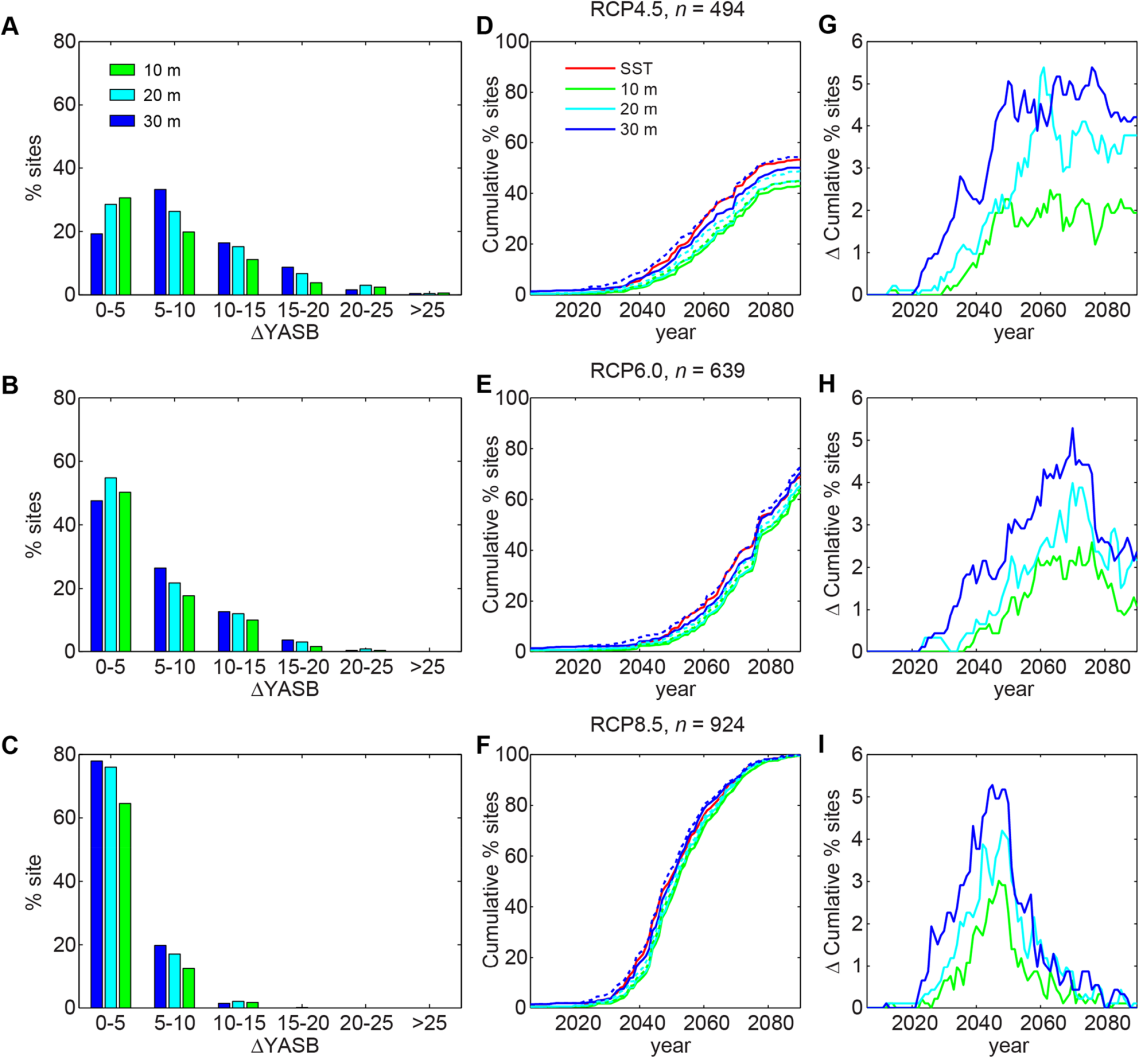
Distribution the number of coral reef sites at 10-, 20-, and 30-m depths experiencing a delay (∆) in projected Year of Annual Severe Bleaching (YASB). Left panels (**A**–**C**): percentage of reef sites for different annual ranges in ∆YASB through 2090. Center panels (**D**–**F**): cumulative percentage of reef sites that reach YASB by each year for reefs at depth and exposed to semidiurnal temperature fluctuations (solid lines) and depth-only (dashed lines) to highlight the effects of the semidiurnal temperature fluctuations; the difference between the lines is the ∆YASB. Right panels (**G**–**I**): Difference in the cumulative percentages that are shown in center panels between the scenarios that only include depth (dashed lines in **D**–**F**) and those that include both depth and semidiurnal temperature fluctuations (solid lines in **D**–**F**). Each row presents a different Representative Concentration Pathway (RCP) scenario: top is RCP4.5, middle is RCP6.0, and bottom is RCP8.5. For all plots, only reef sites that have a corresponding mode-1 internal tide amplitude and also reach YASB due to SST before 2090 are considered (*n* = total number). Semidiurnal temperature fluctuations provide a cooling benefit to corals at 10–20-m depths; however, for 30-m depths, the limited annual temperature range makes the coral highly sensitive to temperature variations. The contribution of semidiurnal fluctuations to cooling is greatest later in the century for the RCP4.5 and 6.5 scenarios, but during the middle of the century for RCP8.5.

Previous studies^3,4,18,19^ have projected the vast majority of reefs to experience annual bleaching by the mid twenty-first century for high emission scenarios such as RCP8.5. Our results indicate that when considering exposure to semidiurnal temperature fluctuations, this prediction does not change, except for a small subset of sites. The thermal benefit (i.e., subsurface cooling causing a delay in YASB) provided by semidiurnal temperature fluctuations changes across the different emission scenarios. Across all water depths, the greatest thermal benefit occurs for the low emission (RCP4.5) than the high emission (RCP8.5) scenario, for the cooling benefit is not enough to offset the additional thermal stress at the highest projected SSTs later in the century (Fig. 3). Across reefs, the most extensive thermal buffering for all emission scenarios is projected for the shallowest (10 m) reef sites. This likely occurs because they have relatively high thermal tolerances due to their proximity to higher surface temperature variability but receive cooling benefits from the subsurface temperature fluctuations. Interestingly, the contribution of semidiurnal fluctuations to cooling is greatest later in the century for the RCP4.5 and 6.5 scenarios, but for RCP8.5 it peaks during the middle of the century, followed by a decline. This is because, as the number of coral reef sites reaching their YASB rapidly increases, even a small ∆YASB during the middle of the century results in a relatively large effect.

**Figure 4:**
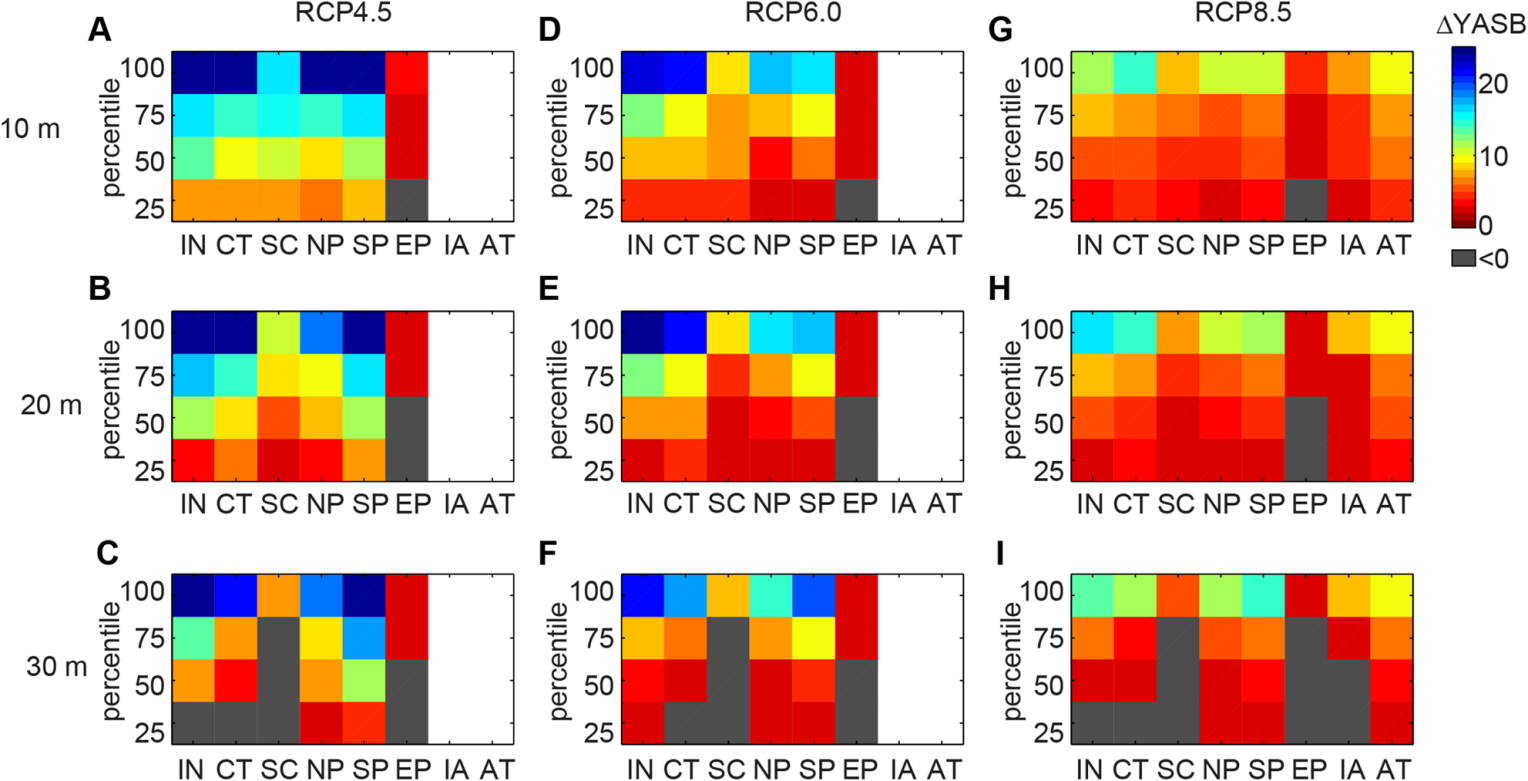
The percentile distribution in delay (∆) in projected Year of Annual Severe Bleaching (YASB) compared to YASB for sea-surface temperature (SST) alone for coral reef sites by depth for different Representative Concentration Pathway (RCP) scenarios, grouped by geographic region. (**A**–**C**) RCP4.5. (**D**–**F**) RCP6.0. (**G**–**I**) RCP8.5. Depths are by row: 10-m (top row), 20-m (middle row), and 30-m (bottom row). For all plots, only reef sites that have a corresponding mode-1 internal tide amplitude and also reach YASB due to SST by 2090, the latest year possible with global climate models, are considered (*n* = total number). The regions are: Indian Ocean (IN; *n* = 80 (RCP4.5), 87 (RCP6.0), 146 (RCP8.5)), Coral Triangle (CT; *n* = 1,300, 156, 220), South China Sea (SC; *n* = 6, 20, 30), North Pacific Ocean (NP; *n* = 107, 136, 156), South Pacific Ocean (SP; *n* = 160, 227, 271), Eastern Pacific (EP; *n* = 9, 8, 17), Intra-Americas Sea (IA; *n* = 0, 2, 73), and Atlantic Ocean (AT; *n* = 2, 8, 8). Gray colors indicate negative values, and in cases where *n* < 5, the region is left blank. See Fig. 1 for the locations of the regions.

Deeper corals experience temperatures cooler than SST, however, the degree of this cooling for a given depth depends on ocean vertical structure and, in particular, the depth of the seasonal thermocline. A shallow seasonal thermocline coupled with large internal wave amplitudes can produce the greatest thermal benefit (cooling). Although the number of coral reef sites in each geographic region varies, the projections generally indicate less thermal benefit to corals from semidiurnal internal tide activity in the Eastern Pacific Ocean, South China Sea, and Intra-Americas Seas, as compared to the Indian Ocean, North and South Pacific Oceans, and Coral Triangle (Fig. 4). The higher benefit in the latter regions is due to a shallow seasonal thermocline coupled with exposure to greater semidiurnal internal tide energy (Fig. 1). For all regions and across all depths, the thermal benefits provided by depth and semidiurnal temperature fluctuations decreases substantially with increasing emission scenario (Fig. 4). The Indian Ocean, Coral Triangle, North Pacific, and South Pacific are the geographic regions with coral reef sites that both have a corresponding internal tide amplitude and an SST YASB before 2091. Of these four regions, the Indian Ocean has the highest percentage of reef sites with delays ≥ 10 yr under all emissions scenarios.

## Discussion

The results presented here demonstrate how the influence of semidiurnal internal tides could act to delay thermally induced bleaching for pockets of reefs across the global ocean. There are a number of other sub-mesoscale processes that also drive upwelling of cooler waters onto coral reefs that are not captured here. As a result, the projections provided here are relatively conservative and likely underrepresent the importance of such subsurface hydrodynamic processes to provide thermal refugia and, thus, provide time to allow corals to adapt, either on their own or via human intervention, to the warming oceans. In addition, it remains unclear how water column structure and subsurface cooling processes, including internal tide energetics, will be modulated by to climate change due to likely changes in surface versus subsurface heating^38,39^ and mixing by projected changes in wind and ocean surface waves^40^.

The Coral Triangle encompasses the majority of sites predicted to have ≥ 10 yr and ≥ 20 yr delays in YASB due to semidiurnal temperature fluctuations alone, particularly in the Indonesian Seas. Satellite surveys indicate the Indonesian Seas have an exceptionally high prevalence of internal waves, due to the large number of interisland sills that separate deeper basins coupled with year-round stratification^41,42^. In addition, the Coral Triangle has the world's highest levels of marine biodiversity and over 500 species of reef-building coral^43^. Under RCP8.5, most of the considered Coral Triangle sites are predicted to reach YASB before 2091, but for corals at 10 m, the combined effects of depth and the semidiurnal temperature fluctuations can delay the onset of annual severe bleaching by 1–2 decades. Thus, the high species diversity, coupled with cooling driven by internal waves, underscores the potential of the Coral Triangle to serve as refugia for corals under increasing SST.

Increasingly, MPAs have been used as a management tool for protecting coral reefs around the globe^44^. The locations chosen for most MPAs were established based on criteria such as specific coral species of concern, coral ecosystem biodiversity, and socio-economic drivers^45^. MPAs have been generally used to address local threats to coral reefs such as overfishing or land-based pollution and not the threat of thermally induced coral bleaching, as increasing global sea temperatures are not something that local managers can control. MPAs will not be protected from increasing ocean temperatures unless a location is chosen that experiences natural thermal buffering. Because thermally induced coral bleaching^2^ is projected to increase in both magnitude and frequency, research can help identify natural refugia from thermal stress to aid the decision-making process^46,47^ of resource managers designating MPAs with the goal to protect and preserve corals, especially if emissions continue to track the high-end RCP8.5 scenario. Identification of such areas would help provide additional time for managers to conduct interventions and coral reefs to adapt.

In order to improve predictions of the magnitude and location of internal tide cooling effects on coral reefs, a number of improvements in our understanding of fine-scale baroclinic processes must occur. For example, the processes, timescales, and spatial scales over which internal tide energy translates from the open ocean to nearshore environments must be better constrained. As the resolution of coupled atmosphere–ocean GCMs used to project future climate improves, they might better capture the effects of climate change on global internal tide energetics and how they drive local-scale cooling of reefs may change in the future. As more detailed observation or models of those processes become available, they could be incorporated into the analyses provided here to better constrain the influence of such processes to buffer increasing ocean temperatures.

The synergistic effects of water-column structure and internal wave energy described here provide an improved understanding of where, and to what extent, internal tidal motions might provide beneficial cooling to coral reefs around the globe for different emission scenarios. The buffering effects of such motions are greater and occur later in the twenty-first century for lower emission scenarios than for the high-end scenario. Incorporation of information on how global carbon emission scenarios may affect these processes that help buffer coral reefs from projected thermally induced bleaching will support the planning and management necessary to protect and preserve coral reefs in the face of climate change.

## Supplementary information

Supplementary Information.

## Data Availability

The datasets analyzed during the current study are included in this published article (and its Supplementary Information files).
