# Supplementary material for: Internal tides can provide thermal refugia that will buffer some coral reefs from future global warming
Source: Sci Rep. 2020 Aug 10;10:13435. doi: 10.1038/s41598-020-70372-9 (PMC7417736; doi:10.1038/s41598-020-70372-9)
Supplement: Supplementary file 1 — Supplementary Information. [file 41598_2020_70372_MOESM1_ESM.docx]

**SUPPLEMENTARY INFORMATION**

**Title:**

Internal tides can provide thermal refugia that will buffer some coral reefs from future global warming

**List of Authors:**

Curt D. Storlazzi^1^, Olivia M. Cheriton^1^, Ruben van Hooidonk^2^, Zhongxiang Zhao^3^, Russell Brainard^4^

^1^ U.S. Geological Survey, Pacific Coastal and Marine Science Center, Santa Cruz, CA 95060, USA

^2^ University of Miami, Cooperative Institute for Marine and Atmospheric Studies, Miami, FL 33149, USA

^3^ University of Washington, Applied Physics Laboratory, Seattle, WA, 98105, USA

^4^ The Red Sea Development Company, The Red Sea Project, Riyahd, Saudi Arabia

**Corresponding Author:**

Curt D. Storlazzi

U.S. Geological Survey, Pacific Coastal and Marine Science Center

2885 Mission Street

Santa Cruz, CA 95060 USA

Office: +1-831-460-7521, Fax: +1-831-427-4748

Email: [cstorlazzi@usgs.gov](mailto:cstorlazzi@usgs.gov)

**Keywords:**

Coral reefs, temperature, bleaching, cooling, internal waves

**Review Disclaimer:**

This draft manuscript is distributed solely for purposes of scientific peer review.  Its content is deliberative and predecisional, so it must not be disclosed or released by reviewers. Because the manuscript has not yet been approved for publication by the U.S. Geological Survey (USGS), it does not represent any official USGS finding or policy.


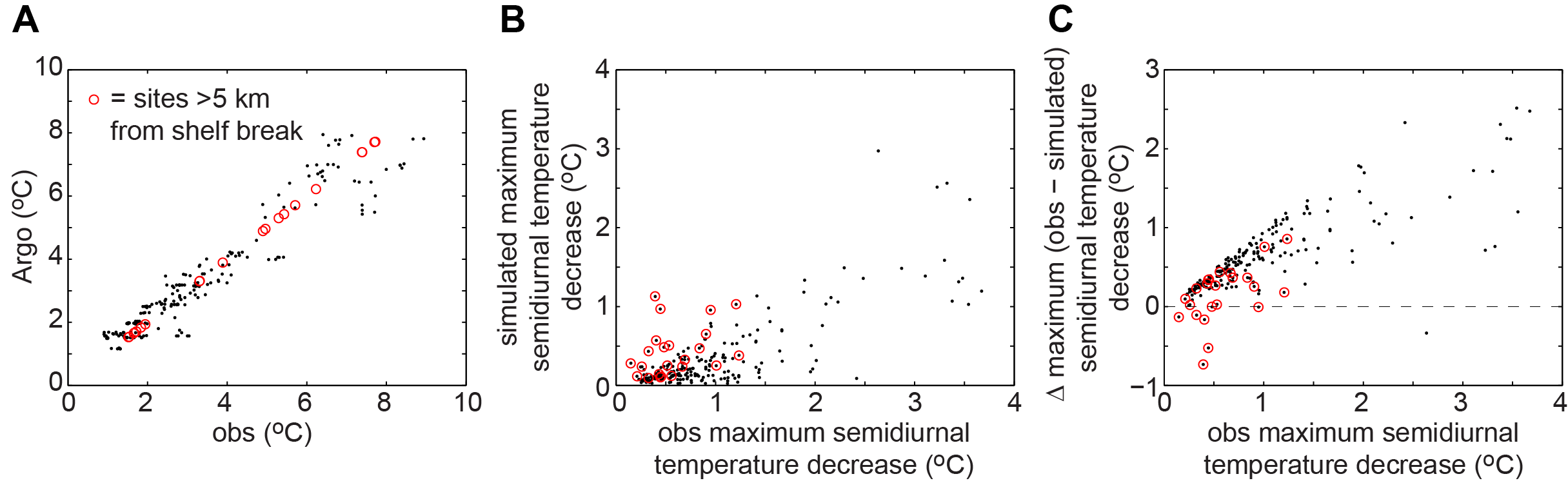


**Supplementary Figure S1.** Comparisons of between the *in situ* measured (‘obs’) temperatures and those from the Argo dataset and the simulated semidiurnal temperatures. Red circles denote sites >5 km inshore of the shelf break excluded from the analyses. (**A**) Seasonal temperature range from the Argo climatology versus the *in situ* data; *r*=0.95, *p*<<0.05, with the regression relationship: Argo = 0.8(obs)+0.6, (σ=0.4). (**B**) Maximum annual temperature decrease due to semidiurnal fluctuations from *in situ* data versus the simulated data; *r*=0.81, *p*<<0.05, with the regression relationship: obs = 1.3(simulated)+0.5, (σ=0.3). With the exception of the broad shelf sites excluded from the model, we generally underestimate the temperature decrease due to semidiurnal temperature fluctuations by ~0.5^o^C. (**C**) Maximum annual temperature decrease due to semidiurnal fluctuations from *in situ* data versus the difference between the in situ and simulated maximum temperature decrease shown in **B**, indicating that the difference in maximum temperature decrease scales with the magnitude of observed maximum temperature decrease; any sites the fall below y=0 are overestimates.


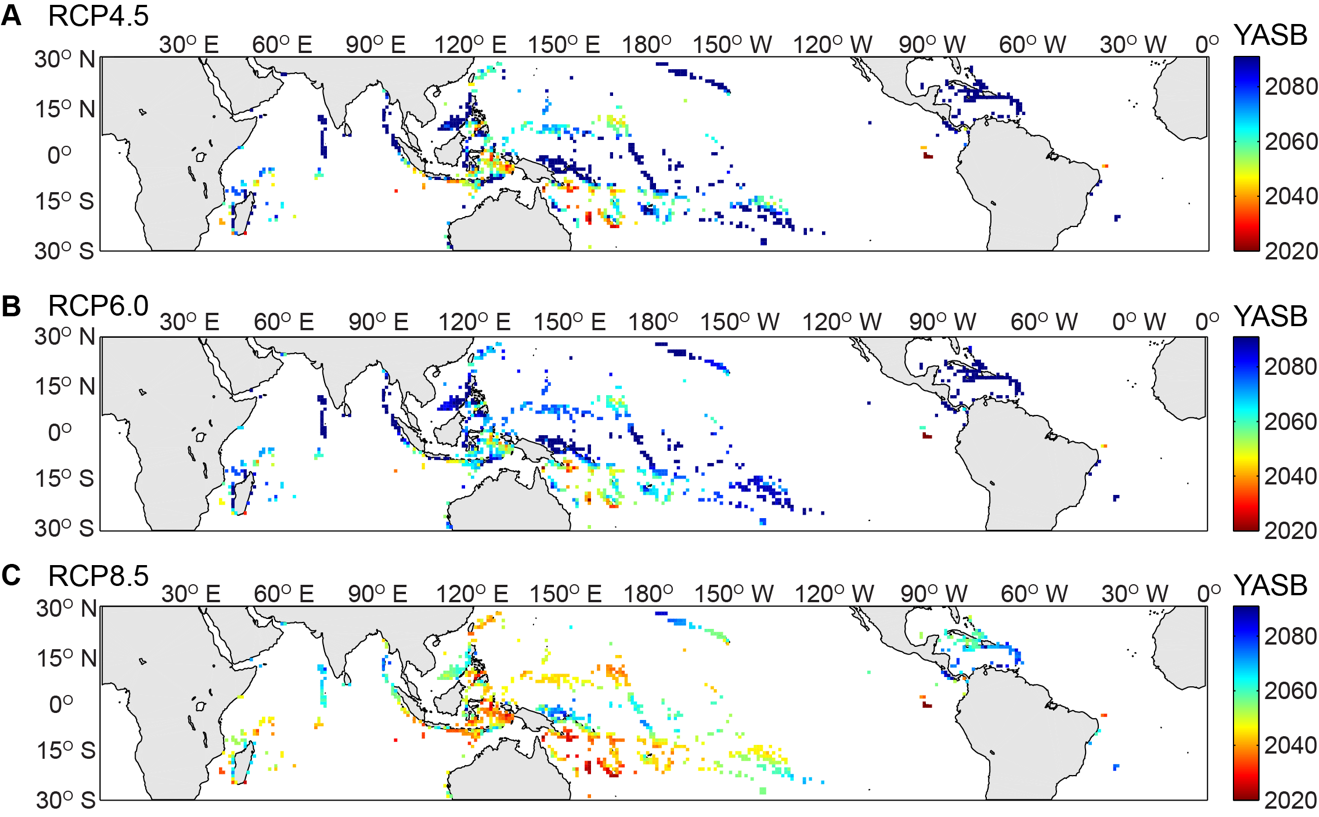


Supplementary Figure S2. Map showing coral reef locations and the projected Year of Annual Severe Bleaching (YASB) for different Representative Concentration Pathway (RCP) scenarios. (A) RCP4.5. (B) RCP6.0. (C) RCP8.5.

Supplementary Table S1. List of in situ water temperature data from subsurface temperature records deployed at coral reef sites. The data are from the NOAA National Coral Reef Monitoring Program and the Coral Reef Research Foundation on Palau.

|  | Site Name | Date range (year-month-day) | Dataset Identifier or web location |
| --- | --- | --- | --- |
| 1 | Hawaiian Archipelago | 2013-07-13 to 2016-09-28 | [gov.noaa.nodc:0162216](https://accession.nodc.noaa.gov/0162216) |
| 2 | Pacific Remote Island Areas | 2012-03-04 to 2015-11-12 | [gov.noaa.nodc:0162217](https://accession.nodc.noaa.gov/0162217) |
| 3 | Wake Island | 2011-03-22 to 2014-03-19 | [gov.noaa.nodc:0162218](https://accession.nodc.noaa.gov/0162218) |
| 4 | Hawaiian Archipelago | 2008-09-20 to 2013-09-14 | [gov.noaa.nodc:0162219](https://accession.nodc.noaa.gov/0162219) |
| 5 | American Samoa | 2012-04-08 to 2013-04-03 | [gov.noaa.nodc:0162220](https://accession.nodc.noaa.gov/0162220) |
| 6 | Marianas Archipelago | 2011-04-09 to 2014-05-06 | [gov.noaa.nodc:0162244](https://accession.nodc.noaa.gov/0162244) |
| 7 | American Samoa | 2012-03-21 to 2015-03-25 | [gov.noaa.nodc:0162246](https://accession.nodc.noaa.gov/0162246) |
| 8 | across the Pacific Ocean | 2001-09-20 to 2012-09-28 | [gov.noaa.nodc:0162471](https://accession.nodc.noaa.gov/0162471) |
| 9 | Florida Reef Tract* | 2013-12-12 to 2016-12-12 | [gov.noaa.nodc:0171795](https://accession.nodc.noaa.gov/0171795) |
| 10 | Florida Keys Reef Tract* | 2013-12-02 to 2016-12-13 | [gov.noaa.nodc:0171796](https://accession.nodc.noaa.gov/0171796) |
| 11 | Palau | 2003-12-01 to 2016-12-31 | http://wtc.coralreefpalau.org/ |

*Manzello, D., Enochs, I., Dutra, E., Morris, J., Kolodziej, G., Jankulak, M., Hendee, J., National Oceanic and Atmospheric Administration, Cooperative Institute for Marine and Atmospheric Studies (2018).
